# Imaging of Sodium MRI for Therapy Evaluation of Brain Metastase with Cyberknife at 7T: A Case Report

**DOI:** 10.7759/cureus.2502

**Published:** 2018-04-18

**Authors:** Lichao Huang, Zihao Zhang, Baolin Qu, Zhiqiang Cui, Yao Wang, Jiwei Li, Jinyuan Wang, Zhentao Zuo, Yan Zhuo, Xinguang Yu, Zhipei Lin, Longsheng Pan

**Affiliations:** 1 Department of Neurosurgery, PLA General Hospital, Beijing, CHN; 2 State Key Laboratory of Brain and Cognitive Science, Institute of Biophysics, Chinese Academy of Sciences; 3 Department of Radiation Oncology, PLA General Hospital, Beijing, CHN; 4 Institute of Biophysics, State Key Laboratory of Brain and Cognitive Science Chinese Academy of Sciences

**Keywords:** sodium mri, brain metastase, cyberknife, tissue sodium concentration (tsc)

## Abstract

Herein we describe the case of an elderly patient who presented with a recent history of impaired vision of the right eye around three months due to brain lesions. He was diagnosed with liver cancer and underwent surgery three months prior. The pathological result is hepatocellular carcinoma. Magnetic resonance imaging (MRI) revealed the diagnosis of brain to be metastatic. The patient selected CyberKnife (Accuray Incorporated, Sunnyvale, USA) radiosurgery for the brain lesion since his physical conditions are not suitable for craniotomy. We adapt the imaging of sodium MRI and proton diffusion mapping at 7T MR system to evaluate the efficacy following CyberKnife early stage treatment. To date, we find the tissue sodium concentration (TSC) changes with the time whereas the proton MRI has no significant change within one month. The time course of sodium concentration in the tumor showed a dramatic increase in the treated brain tumor compared to the pretreatment sodium concentration and 48 hours after stereotactic radiosurgery (SRS), which is correlated to the period of the radiotherapy-induced cellular necrosis. This case demonstrates the possibility of sodium MRI as a biomarker for monitoring early radiotherapy for assessing tumor cellularity.

## Introduction

Stereotactic radiosurgery (SRS) is an established and effective treatment for brain metastases (BM). The proton MRI is used to evaluate the volume of tumor change after radiotherapy by RECIST (Response Evaluation Citeria in Solid Tumors). However, the changes in the level of tumor cells could not be reflected. In contrast, sodium MRI could reveal tumor microenvironment. Schepkin et al. have investigated the changes of sodium signal of rat brain tumor model in response to the chemotherapy [1]. In our study, we investigated the sequence of changes in the sodium MRI signal in the brain metastasis following CyberKnife radiosurgery and the main focus is on the evaluation for the early response after SRS procedure with CyberKnife.

## Case presentation

**Figure 1:**
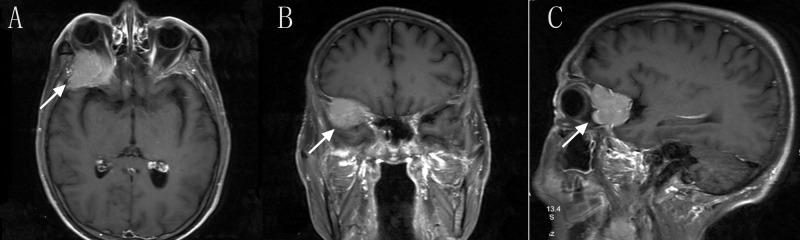
Initial T1 Contrast-enhanced magnetic resonance imaging Axial (A),Coronal (B) and Sagittal (C) T1 Contrast-enhanced MRI imaging revealed an cranio-orbital tumor(arrow).

A 60-year-old man who presented with a recent history of impaired vision of right eye for about 3 months due to brain lesions.  He was diagnosed with liver cancer and underwent surgery three months ago. The pathological result is hepatocellular carcinoma. His right eye showed impaired vision and the proton MRI revealed brain metastasis located in the right orbital part (Figure 1).

**Figure 2:**
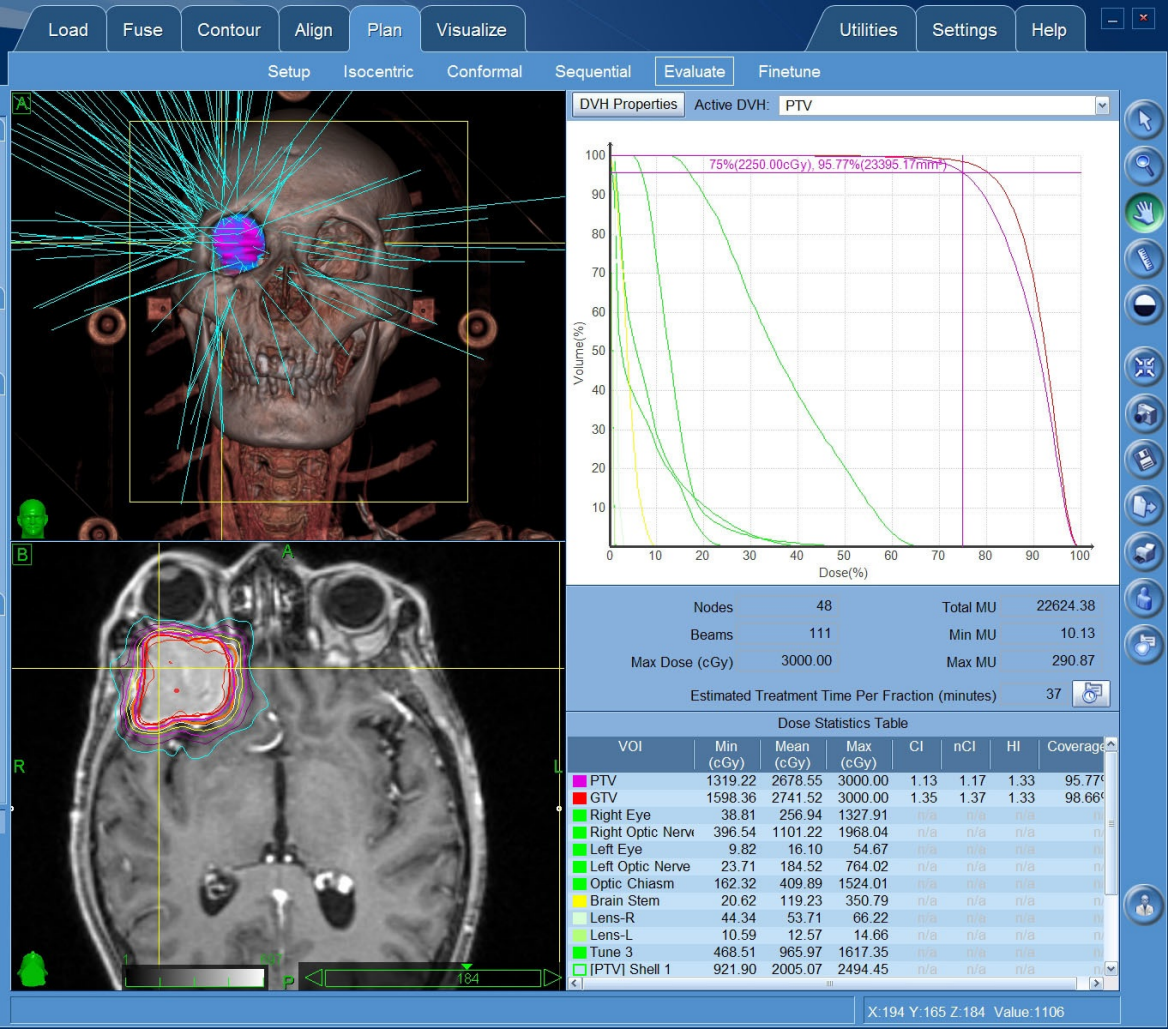
CyberKnife radiosurgery planing

**Figure 3:**
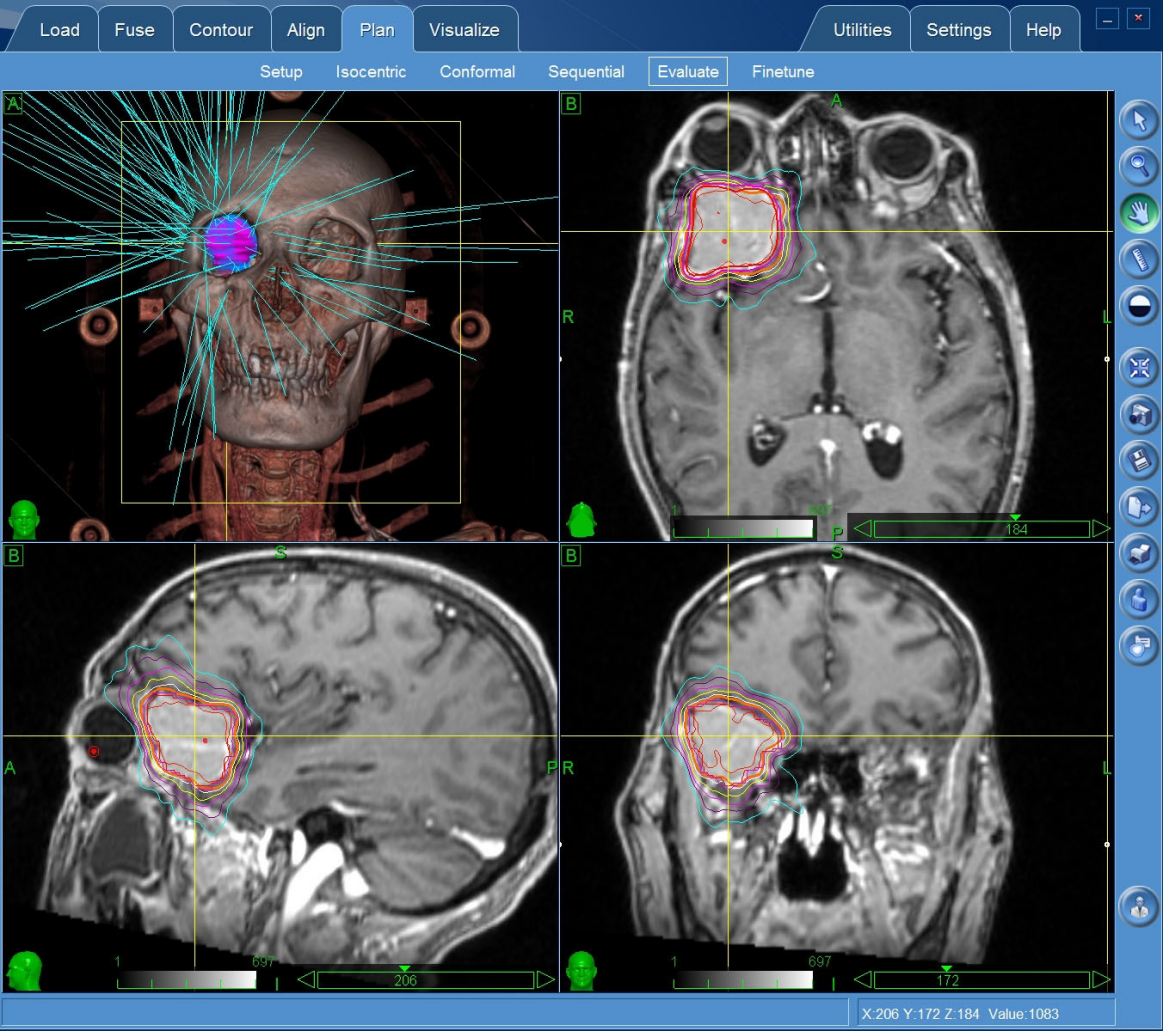
Cyberknife radiosurgery planing

The patient was not suitable for the surgical excision due to his poor physical conditions after liver tumor resection. CyberKnife radiosurgery was delivered at a dose of 22.5 Gy in three fractions (Figure 2-3).

Both sodium and proton MRI (Siemens Magnetom 7T, Erlangen, Germany) were performed periodically at 7T by the time schedule of pre-treatment, 48 hours after SRS, with one week follow up and one month follow up. The scan parameters are listed in Table 1.

**Table 1:** Scan parameters FOV (Field of view) FLAIR (Fluid Attenuated Inversion Recovery) DWI (Diffusion Weighted Imaging)

Scan Parameters			
Sequence	FOV(mm)	Resolution(mm)	Time（min）
T1	224x203x179	0.70x0.70x0.70	5:06
T2	190x199x103	0.26x0.26x2.5	7:28
FlAIR	220x199x117	0.43x0.43x3.0	7:58
DWI	196x196x120	2.0x2.0x2.0	4:48
Sodium	224x224x224	3.5x3.5x3.5	~14:00

**Figure 4:**
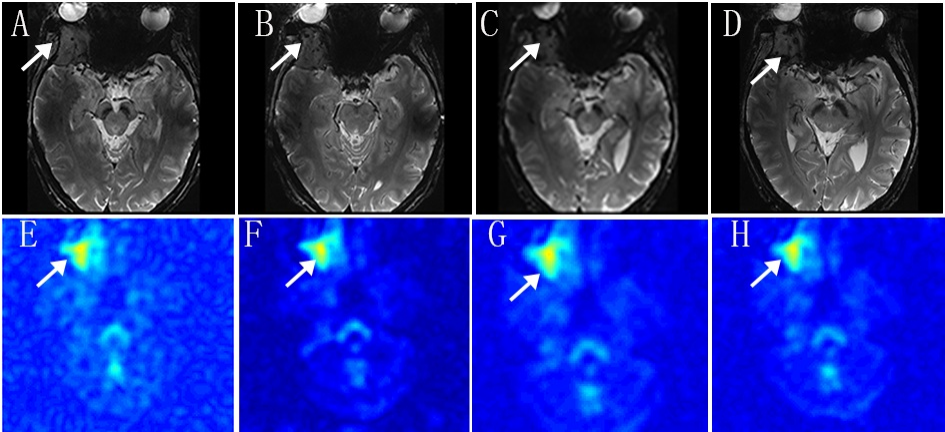
T2-weight imaging and sodium MRI. Axial T2-weight MRI with pretreatment (A), 48 hours after treatment (B), post-treatment of 1 week (C) and post-treatment of 1 month (D). Axial Sodium MRI Images with pretreatment (E)  48 hours after treatment (B), post-treatment of 1 week (C), post-treatment of 1 month (D)

The sodium signal in the tumor is obviously higher than the opposite normal brain tissue within one month after SRS (E-H). However, the T2 magnetic resonance imaging reveals no obvious changes (A-D) (Figure 4).

The quantified sodium signal intensity in MRI was shown in Table 2.

**Table 2:** Sodium MRI Signal Intensity

Scan	untreated	48 hours	one week	one month
Tumor	1599.729244	1619.115858	1551.354553	1703.269578
Ventricle	1195.083046	1188.588231	1226.508085	1233.800309
Ratio	1.338592535	1.362217643	1.26485473	1.380506688

**Figure 5:**
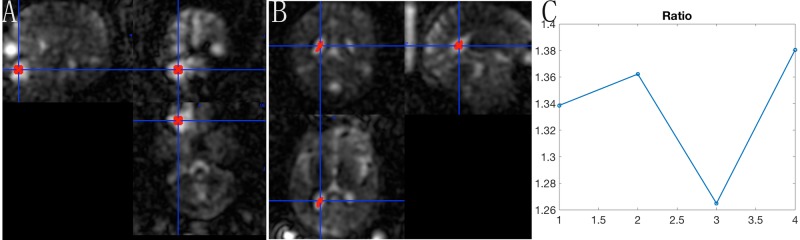
The TSC ratio of tumor and cerebrospinal fluid The sodium signal intensity of ROI (region of interest) located in the center of tumor (A) as well as the sodium signal intensity of CSF in lateral ventricle (B) were quantified. The ratio of sodium signal intensity in tumor and CSF is shown clearly (C).

The time course of sodium signal intensity in the tumor showed a dramatic increase in the treated brain tumor compared to the pretreatment and SRS within 48 hours. And the signal intensity decreased at one week after SRS compared to 48 hours after SRS. However, the increased signal intensity was observed at one month. The TSC ratio of tumor to cerebrospinal fluid (CSF) is much more intuitive (Figure 5).

## Discussion

Stereotactic radiosurgery (SRS) is becoming a recognized, fashionable treatment option for brain metastases with reported excellent local control rates. And the multi-session SRS delivered by CyberKnife is an effective option for the treatment of intraorbital lesions with tumor size shrinkage, pain relieving, and vision preserved. The fractionated scheme applied in our study with a dose of 22.5 Gy in three fractions is based on the indication of SRS.

The therapeutic responses of brain tumors are conventionally assessed by size and contrast enhancement characteristics on CT or MRI images. With the advancement of molecular imaging, biomarkers are adopted with its ability to reflect the early prediction of treatment outcome since cellular changes occur earlier, before tumor shrinkage observed [2]. Sodium ions (23Na+) are vital components in human brains, and the cellular homeostasis process is through coupled exchange with potassium ions K+ between the intra- and extra-cellular compartments by Na+/K+-ATPase (sodium-potassium pump) [3]. Dysregulation of the sodium-potassium pump, or of ATP-dependent processes in the cells will provoke dysregulation of ion homeostasis and therefore leads to an increase of intracellular sodium concentration (TSC) as the gradient cannot be sustained anymore, and furthermore to cell death [4]. These bio scales (TSC) can monitor the spatial distribution of tissue responses to radiation treatment on at least a weekly basis and could be used to guide adaptive radiation treatment for each patient and avoid excessive radiation when no response can be achieved [5]. Our study was to investigate the TSC change before the tumor shrinkage in humans.

Data presented in this our study reveal that the sodium signal in pretreatment tumor is obviously higher than the opposite normal brain tissue. Because the cell membrane depolarization precedes cell division in proliferative neoplastic tissue, leads to an increase in the intracellular sodium concentration and a concomitant rise in the total sodium concentration in the tumor tissue [6]. The first TSC peaks after CyberKnife radiosurgery within 48 hours. Sodium overload may have a strong connection with apoptosis and even initiate apoptosis itself [7]. We consider that the first TSC peak in 48 hours is related to radiotherapy-induced apoptosis. The second TSC peak in one month may be related to tumor recurrence, however, further pathological results could not be obtained. Further MRI examination will be performed by image findings.

The sodium MRI offers complementary information which could be quantitatively measured and it is non-invasive. Even though the clinical value of sodium MRI as a complement to proton MRI or other imaging modalities such as PET and CT is still under investigation [8], our study shows the clinical value with due to the biochemical information provided.

## Conclusions

In conclusion, we present a case of complicated brain metastatic treated with CyberKnife radiosurgery. The case demonstrates the possibility of sodium MRI as a biomarker for monitoring early radiotherapy for assessing tumor cellularity. The noninvasive and rapid feedback may be used to guide the management of brain tumors.
